# MicroRNA-34c Enhances Murine Male Germ Cell Apoptosis through Targeting ATF1

**DOI:** 10.1371/journal.pone.0033861

**Published:** 2012-03-30

**Authors:** Xiaoxuan Liang, Doudou Zhou, Chao Wei, Haoshu Luo, Jiali Liu, Rui Fu, Sheng Cui

**Affiliations:** State Key Laboratory of Agrobiotechnology, College of Biological Sciences, China Agricultural University, Beijing, People's Republic of China; Ospedale Pediatrico Bambino Gesu', Italy

## Abstract

**Background:**

MicroRNAs (miRNAs) play vital regulatory roles in many cellular processes. The expression of miRNA (miR)-34c is highly enriched in adult mouse testis, but its roles and underlying mechanisms of action are not well understood.

**Methodology/Principal Findings:**

In the present study, we show that miR-34c is detected in mouse pachytene spermatocytes and continues to be highly expressed in spermatids. To explore the specific functions of miR-34c, we have established an *in vivo* model by transfecting miR-34c inhibitors into primary spermatocytes to study the loss-of-function of miR-34c. The results show that silencing of miR-34c significantly increases the Bcl-2/Bax ratio and prevents germ cell from apoptosis induced by deprivation of testosterone. Moreover, ectopic expression of the miR-34c in GC-2 cell trigger the cell apoptosis with a decreased Bcl-2/Bax ratio and miR-34c inhibition lead to a low spontaneous apoptotic ratio and an increased Bcl-2/Bax ratio. Furthermore, ectopic expression of miR-34c reduces ATF1 protein expression without affecting ATF1 mRNA level via directly binding to ATF1's 3′UTR, indicating that ATF1 is one of miR-34c's target genes. Meanwhile, the knockdown of ATF1 significantly decreases the Bcl-2/Bax ratio and triggers GC-2 cell apoptosis. Inhibition of miR-34c does not decrease the GC-2 cell apoptosis ratio in ATF1 knockdown cells.

**Conclusions/Significance:**

Our study shows for the first time that miR-34c functions, at least partially, by targeting the ATF1 gene in germ cell apoptosis, providing a novel mechanism with involvement of miRNA in the regulation of germ cell apoptosis.

## Introduction

In mammals, the differentiation of germ cells into spermatozoa occurs in the seminiferous epithelium of the testis and is highly regulated by extrinsic and intrinsic factors. Small RNAs, including small interfering RNAs (siRNAs), piwi-interacting RNAs (piRNAs) and microRNAs(miRNAs), are involved in both somatic and germ line lineages in a broad range of eukaryotic species by regulating mRNA transcription, stability, and translation [1], [2], [3], [4]. MiRNAs are evolutionarily conserved and regulate protein expression by targeting the mRNA of protein-coding genes.

The pre-miRNA hairpin is cleaved by Dicer, the RNase III enzyme. Dicer conditional knockout mice exhibit infertility, indicating that miRNAs are mechanistically involved in the mammalian spermatogenesis [5], [6], [7].

A number of studies have shown that miR-34c is implicated in the control of the cell cycle, senescence, and apoptosis [8], [9], [10], [11], [12], [13], [14], [15], [16], [17]. Most miRNAs exhibit a tissue-specific expression pattern, and recent studies have reported that miR-34c is preferentially expressed in the mouse testis [18], [19], [20], [21]. MiR-34c is largely p53 independent and is involved in late spermatogenesis in mouse testis [18]. However, the specific function of miR-34c in germ cell is not yet clear.

Activating transcription factor 1 (ATF1), which constitutes a subfamily of the basic leucine zipper transcription factors, mediates the transcriptional response of various extracellular signals and it is involved in cell viability and cell transformation [22], [23], [24], [25]. In human clear cell sarcoma, the ATF1 gene seems to be responsible for maintaining tumor viability [26]. ATF1 also maintains cell viability during early embryonic development [25]. In the testis, ATF1 has been shown to be expressed in spermatocytes, but its function is not clear.

In this study, we find that inhibition of miR-34c prevents murine male germ cell apoptosis through targeting ATF1. The current report unveils the pro-apoptotic activity of miR-34c in male mouse reproductive system.

## Results

### MiR-34c Expression in the developing mouse testis

**Figure 1:**
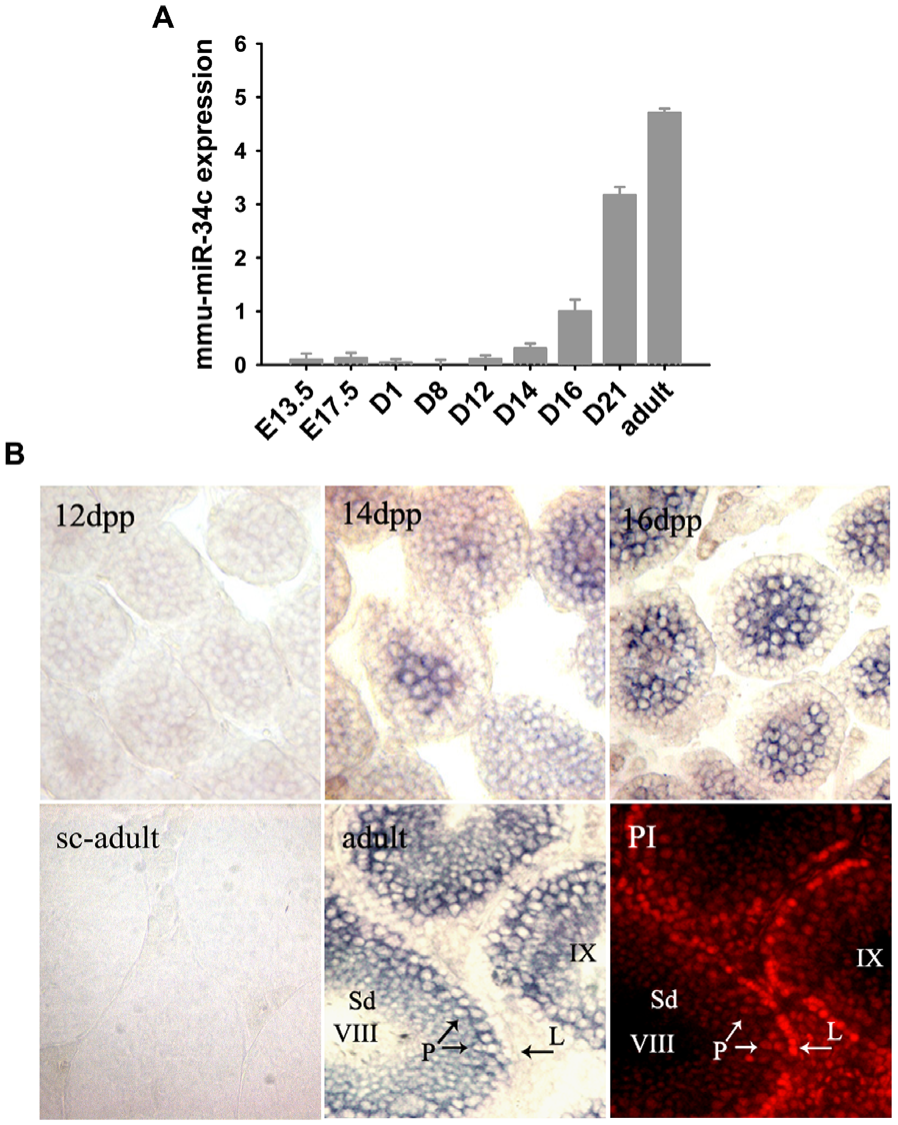
MiR-34c expression is dynamic in immature and adult mouse testes. (A) Real-time PCR for miR-34c. (B) Localization of miR-34c in mouse testis at different development stages using LNA *in situ* hybridization. The first five pictures are 12 dpp-adult ISH of miR-34c and a scrambled probe control (sc-adult). The last picture is a PI staining (red) of cell nuclei of the adult section. All the panels are shown at the same magnifications. L - Leptotene spermatocytes; P - Pachytene spermatocytes; Sd - Spermatids. Data were shown as mean ± S.E.M of three samples for each group.

In order to identify the functions of miR-34c in the mouse testis, we initially examined miR-34c expression in the developing testis by real-time PCR and *in situ* hybridization (ISH). The real-time PCR results showed that testis miR-34c was expressed at very low levels from E13.5 to 12 dpp, after which testis miR-34c expression levels sharply increased and persisted until the adult mouse (Fig. 1A).

We then localized miR-34c expression in the developing testis by using a digoxingenin-labeled locked nucleic acid (LNA) probe during 12 dpp, 14 dpp, 16 dpp and in the adult mouse testis. A scrambled probe was used as a negative control. The miR-34c ISH signal was not detected from E13.5 to 12 dpp (data not shown). The ISH signal for miR-34c initially appeared in pachytene spermatocytes at 14 dpp (Fig. 1B). In 16 dpp testis, the positive signal was much stronger and was distributed in every seminiferous tubule. The cause of this is that the pachytene spermatocytes ratio increases rapidly in seminiferous tubule compared to 14 dpp. In the adult mouse testis, the hybridization signal for miR-34c was detected in pachytene spermatocytes and round spermatids. Propidium iodide (PI) staining was used to identify the stages of the seminiferous epithelium cycle (Roman numbers). These results were consistent with previous studies [18].

### Silencing of miR-34c resulted in resistance to flutamide induced germ cell apoptosis

**Figure 2:**
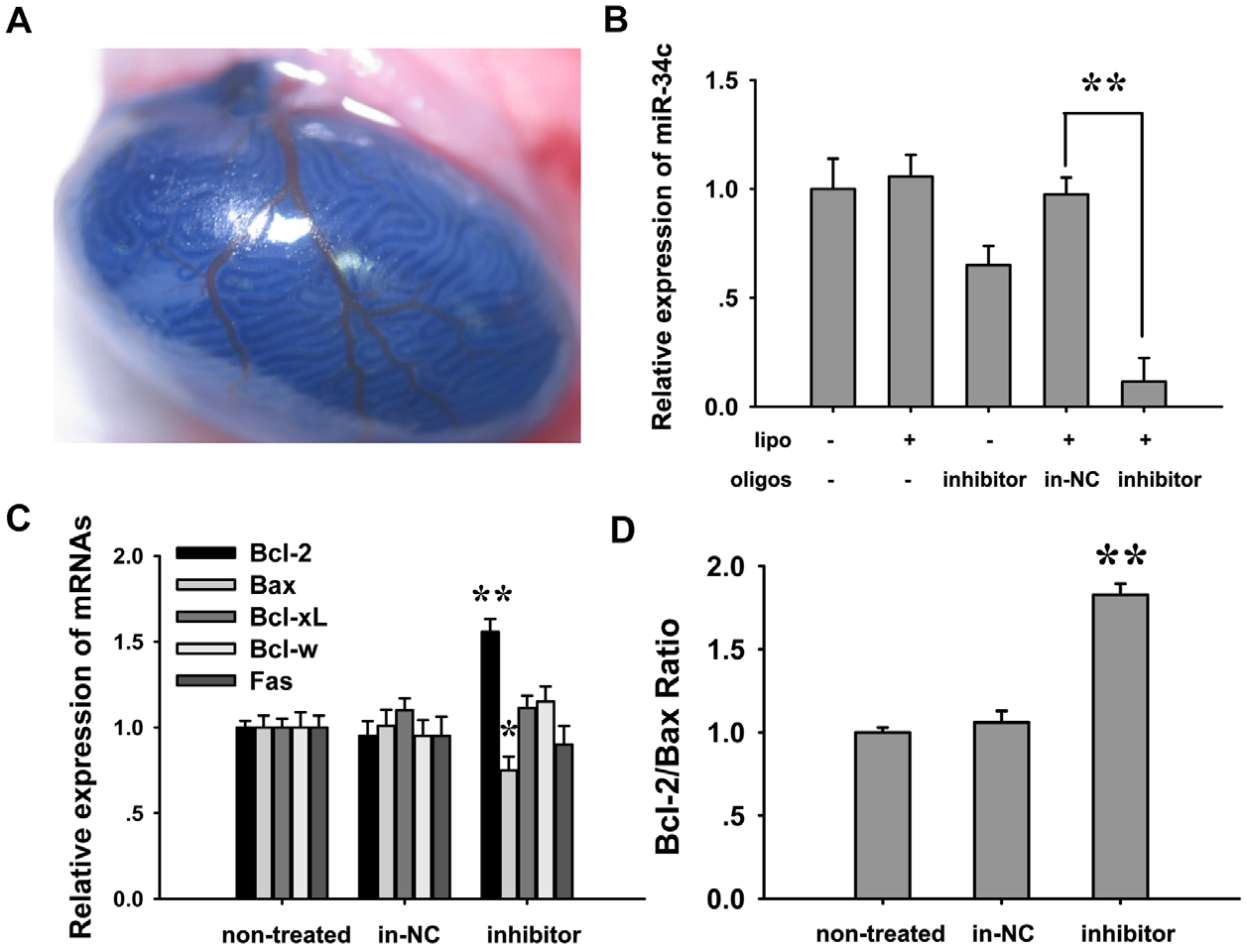
The effects of miR-34c inhibition on Fas, Bcl-2, Bax mRNA expressions and Bcl-2/Bax ratio. (A) MiRNA inhibitor with Lipofectamine™ 2000 injected into the seminiferous tubule of 14 dpp mouse testis. 0.4% Trypan blue (10 fold more than needed for experimental concentrations and used in order to take a clear picture) was added to transfection mix. (B) Real-time PCR analysis two days after injection. (lipo: lipofectamine™; inhibitor: miR-34c inhibitor; in-NC: miRNA inhibitor nonsense control. (C) Apoptosis-related genes were tested by real-time PCR after miR-34c knockdown. (D) Bcl-2/Bax ratio increased after miR-34c inhibition. Each bar presents the mean ± S.E.M of 6 testes from different mice. (*P<0.05, **P<0.01).

In order to identify the function of miR-34c in germ cells, we initially inhibited miR-34c by transfecting a miR-34c inhibitor, which was injected into the seminiferous tubule of 14 dpp testis. 0.04% Trypan blue was added in the complex as an indicator (Fig. 2A). To measure the inhibiting efficiency, the testes (n≥6) were collected and miR-34c levels were detected by real-time PCR at 48 hours (16 dpp) after transfection. The results showed that the inhibiting efficiency was up to 85%, compared to the control (Fig. 2B).

Both the intrinsic pathway and the extrinsic pathway are responsible for germ cell apoptosis [27], [28], [29]. The intrinsic apoptotic pathway hinges on the balance of activity between pro- and anti-apoptotic members of the Bcl-2 superfamily, and low Bcl-2/Bax ratio was a characteristic for apoptotic sensitive cells [30]. We thus measured the effects of miR-34c inhibition on these apoptosis related genes expression. The results showed that the local miR-34c inhibition significantly increased Bcl-2 expression and decreased Bax expression (Fig. 2C), which resulted in the rise of the Bcl-2/Bax ratio (Fig. 2D). However, Fas, acting as a signing molecule related to apoptosis, did not change after silencing miR-34c. These findings suggest that miR-34c could enhance germ cell apoptosis via the intrinsic pathway of cell apoptosis.

**Figure 3:**
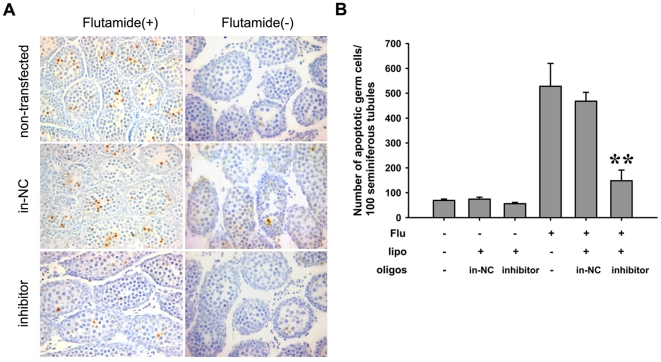
MiR-34c knock-down enhances resistance to cell apoptosis *in vivo*. (A) *In situ* DNA TUNEL labeling after flutamide induction in different treatment groups. Non-transfected: no injection testis; in-NC: inhibitor NC transfection with Lipofectamine 2000 testis as a negative control; inhibitor: miR-34c inhibitor transfection with Lipofectamine 2000 testis. (B) Number of apoptotic germ cells per 100 seminiferous tubules in different treatment group. At least 500 seminiferous tubules were counted for every testis. Each bar presents the mean ± S.E.M of 6 testes from different mice. (*P<0.05, **P<0.01).

To confirm the pro-apoptotic effect of miR-34c in the mouse testis, we used an animal model as reported by Mauduit *et al* [31] to induce apoptosis by intraperitoneal injection of flutamide, an antagonist of androgen. After 24 h treatment with 10 mg/kg, flutamide, the miR-34c knockdown was carried out by injecting the miR-34c inhibitor. Cell apoptosis was detected by *in situ* DNA TUNEL labeling. The TUNEL-positive cells were observed in the seminiferous tubule (Fig. 3A), and in the flutamide treated groups, apoptotic spermatocytes in the miR-34c knockdown testis were much fewer than that in the non-transfected control or in the in-NC control group. For statistical analysis, we counted apoptotic cells in more than 500 seminiferous tubules per testis (Fig. 3B). In the flutamide treated groups, miR-34c knock-down testes had roughly two thirds less apoptotic cells than that in the controls (Fig. 3B). These data indicate that the miR-34c knock-down enhances the resistance of germ cell apoptosis induced by flutamide.

**Figure 4:**
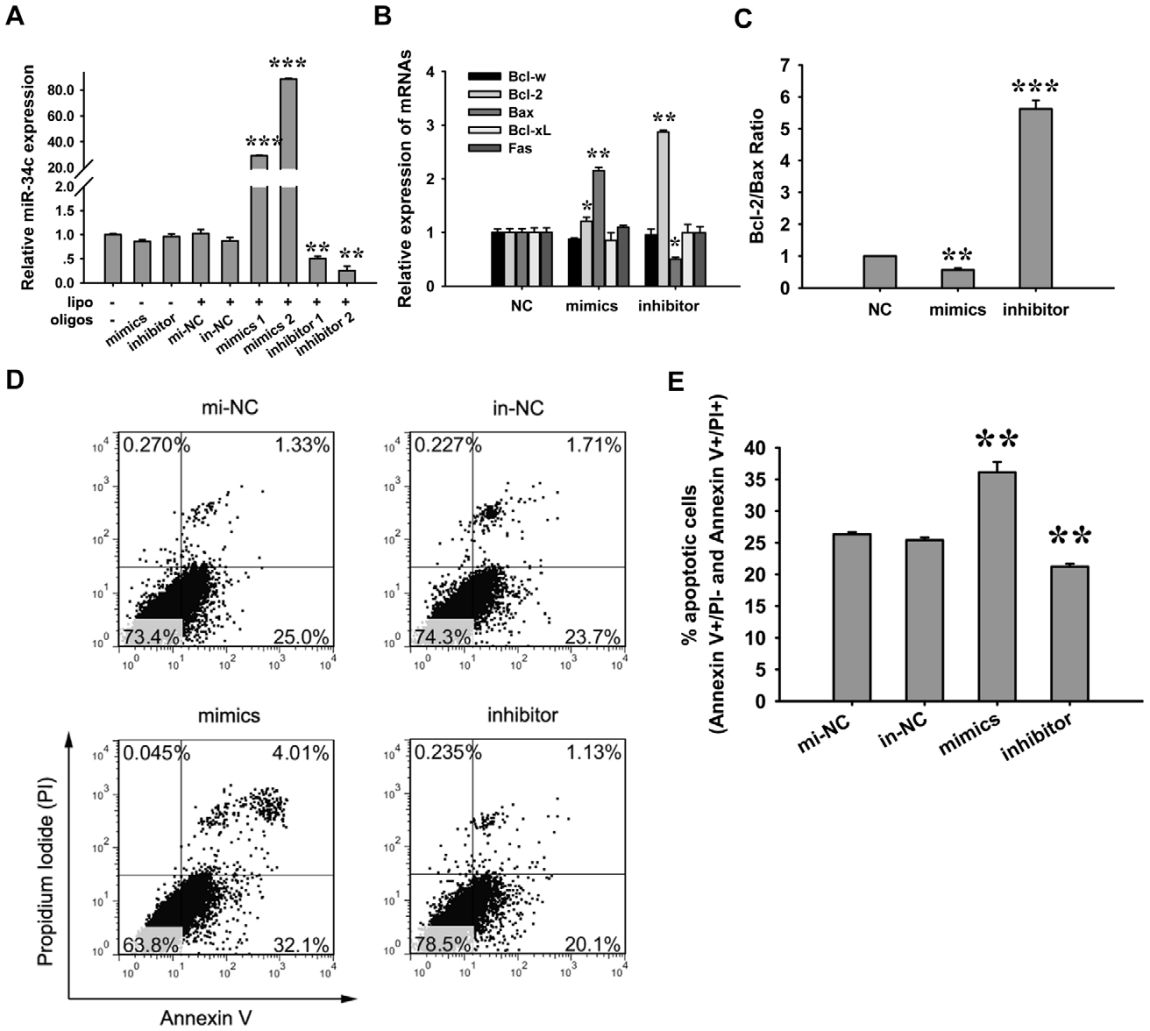
Effects of miR-34c on GC-2 cell apoptosis. (A) Overexpression and inhibition efficiency of miR-34c mimics or inhibitor, transfected into GC-2 cells after 24 h. Non-treated: no transfection control; mi-NC or in-NC: mimics NC or inhibitor NC transfection with Lipofectamine 2000 as a strict control; mimics 1: 20 nM mimics as a final transfection concentration; mimics 2: 30 nM as a final transfection concentration; inhibitor 1 and inhibitor 2: similar to mimics, 20 nM and 30 nM inhibitor were transfected respectively. (B) Apoptosis-related genes expression after mimics or inhibitor transfection. NC, mi-NC or in-NC as mimics or inhibitor control; Mimics L or inhibitor L: 20 nM mimics or inhibitors were transfected into GC-2 cells. (C) The bcl-2/bax ratio changes when miR-34c is overexpressed or inhibited. (D) GC-2 cell apoptosis analysis by Annexin V/Propidium Iodide staining. (E) The summary of GC-2 cell apoptotic ratio after miR-34c overexpression or inhibition. Each bar presents the mean ± S.E.M from three samples for each group. (**P<0.01, ***P<0.001).

To further confirm the pro-apoptotic effect of miR-34c on spermatocytes apoptosis, we transfected miR-34c mimics and inhibitors into the GC-2 cell line. GC-2 cell is a spermatocytes-like cell line and was originally created by Hofmann *et al*. to study the process of germ cell differentiation and apoptosis [32], [33]. The efficiencies of miR-34c over-expression and inhibition were 30∼80 fold and 25%∼40% respectively, for different doses of transfection as analyzed by real-time PCR (Fig. 4A). We then measured Bax and Bcl-2 expression by real-time PCR. MiR-34c over-expression resulted in a sharp increasing of Bax mRNA level compared to the nonsense control. Conversely, the silencing of miR-34c led to high levels of Bcl-2 (Fig. 4B). Bcl-2/Bax ratio decreased after treatment with miR-34c mimics, whereas the Bcl-2/Bax ratio increased once miR-34c was inhibited (Fig. 4C). Similar to the *in vivo* experiment results, miR-34c over-expression promoted cell apoptosis (Fig. 4D–E), whereas miR-34c inhibition resulted in the resistance to cell apoptosis.

### ATF1 is a direct target of miR-34c

**Figure 5:**
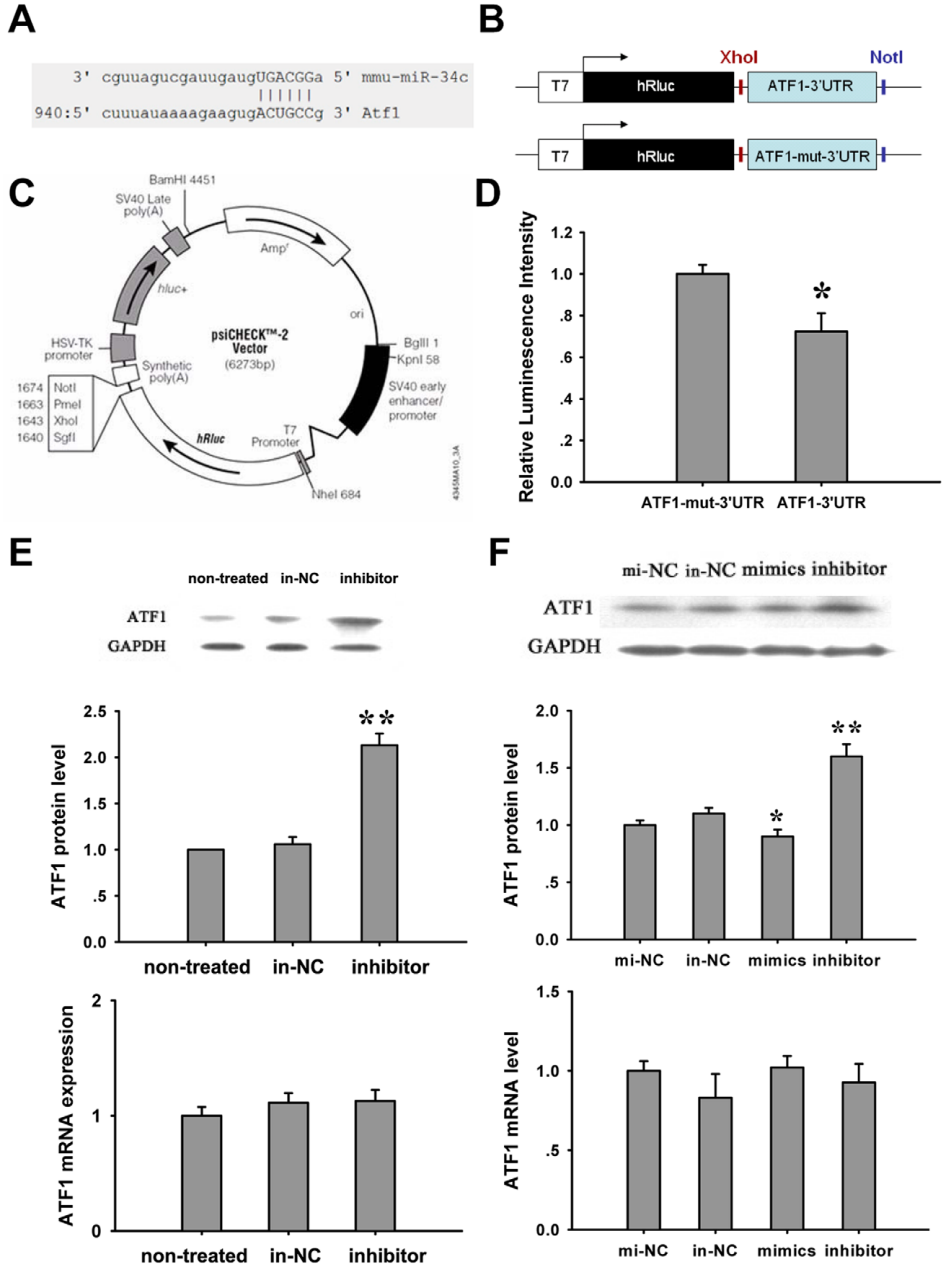
MiR-34c binds to ATF1-3′UTR which regulates its protein expression. (A) The predicted miR-34c binding site in ATF1-3′UTR from www.miRNA.org. (B) Schematic of inserted ATF1-3′UTRs sequences. (C) psi-CHECKTM-2 reporter vector map. (D) Relative luminescence intensity detected by Modulus™II microplate multimode reader after miR-34c mimics and dual-luciferase vector were co-transfected into 293T cells. (E) ATF1 mRNA and protein levels were analyzed by real-time PCR and Western blot after miR-34c inhibition *in vivo*. (F) ATF1 mRNA and protein levels *in vitro* in a GC-2 cell culture after miR-34c overexpression or inhibition. Each bar presents the mean ± S.E.M from three samples for each group. (*P<0.05, **P<0.01).

In order to study the mechanism of miR-34c influencing spermatocytes apoptosis, we computationally predicted that ATF1 was the candidate of miR-34c targeting genes from www.miRNA.org (Fig. 5A). ATF1 expression in pachytene spermatocytes has been reported [25]. The predicted binding site, 3′UTR of ATF1 was then inserted downstream from the Renilla luciferase coding region in the reporter vector (Table 1, Fig. 5.B–C). 3′UTR of ATF1 with six mutated nucleic acids in the seed sequence was used as a negative control (Table 1, Fig. 5B). Each reporter construct was separately co-transfected into 293T cells with the miR-34c mimicking molecules. Compared to the mut-ATF1-3′UTR control, the luciferase activity declined by about 27.7% after transfection with miR-34c mimics and ATF1-3′UTR reporter vector (Fig. 5D). Further, ATF1 mRNA and protein levels were analyzed by real-time PCR and Western blot after miR-34c inhibition *in vivo*. ATF1 protein level was significantly increased after miR-34c inhibition (Fig. 5E). Moreover, we obtained similar results when we transfected miR-34c mimics or inhibitors to GC-2 cells *in vitro*. MiR-34c inhibition showed a remarkable increasing protein level of ATF1, although the ATF1 mRNA level did not change (Fig. 5F). These suggest that miR-34c directly regulates ATF1 protein expression through its binding to the ATF1-3′UTR region.

**Table 1:** The ATF1-3′UTR sequences containing the miR-34c binding site were constructed into a psi-CHECK 2 vector.

*ATF1-3′UTR*	CTCGAGCCAAAGCTTTCCTGAGGGGTTTTATAATCATCTTACCGTAAGGTTTGTCAGGTTCTAAAACAGTTACTGAGGCAATCTTACATTGAGACTATTGTGTAATGTAGCCCATGGTACCTTTATAAAAGAAGTGACTGCCGATATATTTTTATAGTGAATCTTTATAAATTCTAATGTTGAGTTTTTAATGATTATTTTAAATGTTTATATAGTTTGTTTAGCAAAAAAGCGGCCGC
*Mut-ATF1-3′UTR*	CTCGAGCCAAAGCTTTCCTGAGGGGTTTTATAATCATCTTACCGTAAGGTTTGTCAGGTTCTAAAACAGTTACTGAGGCAATCTTACATTGAGACTATTGTGTAATGTAGCCCATGGTACCTTTATAAAAGAAGTG**CCGTCA**GATATATTTTTATAGTGAATCTTTATAAATTCTAATGTTGAGTTTTTAATGATTATTTTAAATGTTTATATAGTTTGTTTAGCAAAAAAGCGGCCGC

The nucleic acids in frame are the binding site of miR-34c. The nucleic acids in bold are the seed binding site mutated which was used as the Mut-ATF1 control.

### MiR-34c regulates Bcl-2/Bax ratio and germ cell apoptosis by targeting ATF1

**Figure 6:**
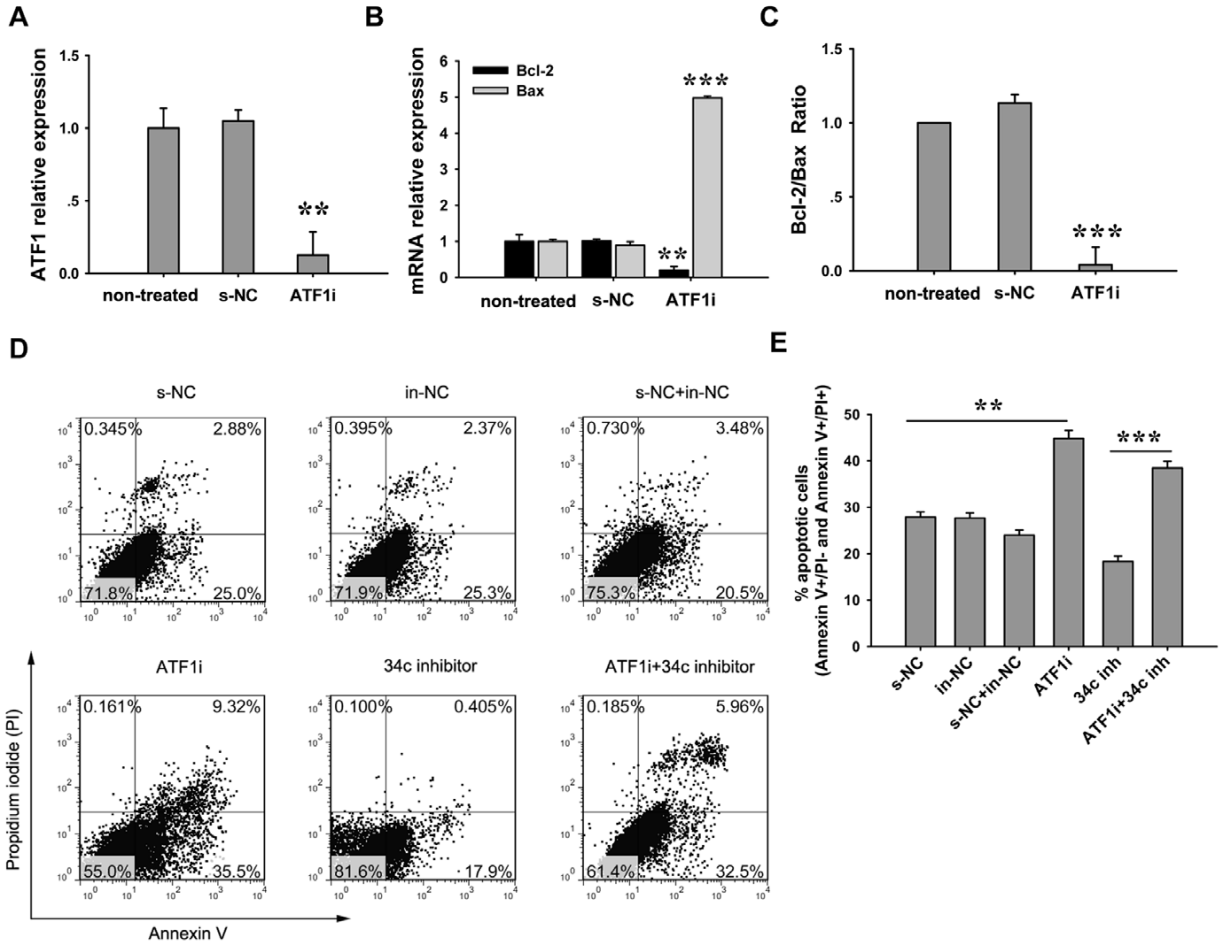
ATF1 regulates GC-2 cell apoptosis and changes the bcl-2/bax ratio. (A) ATF1 siRNA transfected into GC-2 cells to silence its mRNA expression. Knockdown efficiency was analyzed by real-time PCR. Non-treated: non-transfection group; s-NC: stable NC transfected group as a negative control; ATF1i: ATF1 mRNA interference group. (B) Bcl-2 mRNA decreased and Bax mRNA increased significantly after ATF1i. (C) The Bcl-2/bax ratio was reduced very significantly in ATF1i group. (D) GC-2 cell apoptosis analysis by Annexin V/Propidium Iodide staining after ATF1 knockdown or dual-inhibition of ATF1 and miR-34c. (E) The summary of GC-2 cell apoptotic ratio. Each bar presents the mean ± S.E.M from three samples for each group. (**P<0.01, ***P<0.001).

In order to confirm that the miR-34c targeting of ATF1 affects germ cell apoptosis, siRNA molecules of ATF1 were designed to inhibit ATF1 expression. RNAi efficiency analyzed using real-time PCR showed up to 87.4% inhibition (Fig. 6A). Apparently, the ATF1 knockdown significantly up-regulated bax expression and at the same time reduced Bcl-2 expression (Fig. 6B). This resulted in a sharp reduction of the Bcl-2/Bax ratio (Fig. 6C). Similarly, silencing ATF1 showed an increased GC-2 cell apoptosis ratio analyzed by FCM (Fig. 6D). This indicates that the anti-apoptotic activity of ATF1 is regulated through Bcl-2/Bax ratio.

In addition, ATF1 siRNA and miR-34c were co-transfected into GC-2 cells. Compared to the negative control (24∼28%), silencing miR-34c reduced the GC-2 cell apoptosis ratio (∼18.3%) and the knockdown of ATF1 increased the ratio (∼44.82%) (Fig. 6D–E). These results give us important reminding that both miR-34c and ATF1 may be involved in regulating germ cell apoptosis.

## Discussion

The current study reveals that miR-34c regulates germ cell apoptosis in the murine testis. We have for the first time demonstrated that miR-34c mediates posttranscriptional regulation of ATF1 expression to control germ cell viability through an intrinsic pathway of apoptosis. These findings specifically show the pro-apoptotic activity of miR-34c in mouse testis.

Recent studies have shown that miRNAs are evolutionarily conserved and regulate protein expression by either targeting mRNAs to direct their post-transcriptional repression or by regulating translation. MiR-34c is a highly conserved miRNA that is abundantly expressed in mature testis as compared to immature testis, in primates and rodents [19], [34]. We conducted a detailed study of miR-34c expression in the course of testicular development using real-time PCR and *in situ* hybridization. MiR-34c began to be expressed from 14 dpp, in which germ cells have just developed to the pachytene stage [35], [36], and it was continuously expressed during the entire germline differentiation. The fact that miR-34c is not expressed in Sertoli cells and Leydig cells, indicates that miR-34c may play an important role in male spermatogenesis.

It is well documented that in many cancer cells miR-34c is a pro-apoptotic gene that is regulated by the tumor suppressor protein p53 and is frequently observed to be epigenetically silenced in cancers [11], [37], [38]. However, in mouse testis, miR-34c is p53 independent [18]. Ectopic expression of miR-34c in several cell lines resulted in up-regulation of germ cell-specific genes [18]. The specific function of miR-34c in germline differentiation aroused our interest. By transfecting cells, we found that excess expression of miR-34c promotes GC-2 cell apoptosis with an up-regulation of Bax and a down-regulation of Bcl-2 *in vitro*. In contrast, miR-34c inhibition resulted in a reduced GC-2 cells spontaneous apoptosis rate, with an increased expression of Bcl-2 and a decreased expression of Bax. Furthermore, the transient transfection of the miR-34c inhibitor resulted in a resistance to flutamide induced germ cell apoptosis *in vivo*, with an up-regulation of Bcl-2 and a down-regulation of Bax, confirming the pro-apoptotic activity of miR-34c.

Germ cell death is visible during normal spermatogenesis in mammals and plays a pivotal role in sperm output. Testosterone is important for survival of pachytene spermatocytes and is essential for the process of spermatids elongation. Studies showed that an acute degeneration of pachytene spermatocytes and a loss of round spermatids occur when testosterone is deprived [39], [40], [41]. The high expression of miR-34c in pachytene spermatocytes and round spermatids may be responsible for the determination of hormone-dependent survival of germ cells in testis. Furthermore, it has been demonstrated that both the intrinsic pathway and the extrinsic pathway are responsible for germ cell apoptosis [40], [42], [43], [44], [45], [46]. However, miR-34c does not affect the expression of Fas in both *in vivo* and *in vitro* studies, which indicates that miR-34c may regulate germ cell apoptosis through the intrinsic pathway. These results show for the first time that the pro-apoptotic activity of miR-34c in mediating apoptosis was conducted by regulating the Bcl-2/Bax ratio in the male reproductive system.

Effective inhibition of mRNA translation by miRNAs is known to be mediated by the specific site of 3′UTRs. ATF1 3′UTR has a miR-34c binding sequence. ATF1 is a CREB family protein that mediates signals essential for maintaining cell viability and early mouse development [25], [47], [48]. ATF1 forms complexes with CREB by binding to the Bcl-2 promoter region thus promoting the expression of bcl-2 in MCF-7 cells and B cells [49], [50]. Moreover, ATF1 is highly expressed in spermatocytes of the pachytene stage in testis while the CREB protein is found only in Sertoli cells [25], indicating that ATF1 may play a role in spermatogenesis but the specific function of ATF1 in germ cells has not been studied. Here we demonstrated that ATF1 is a direct target gene of miR-34c. MiR-34c represses luciferase reporter gene expression by targeting the 3′-UTR binding site of ATF1 *in vitro*, suggesting a direct interaction between miR-34c and 3′-UTR of ATF1. Furthermore, our study has demonstrated that ATF1 could regulate the expressions of Bcl-2 and Bax in the spermatocyte cell line GC-2, and the knockdown of ATF1 results in a sharp reduction of the Bcl-2/Bax ratio, thus promoting GC-2 cell apoptosis. These results indicate that ATF1 may be key in regulating miR-34c's effect on the expressions of Bcl-2 and Bax. In addition, ATF1 protein expression level is specifically upregulated by silencing miR-34c at 16 dpp testes *in vivo* as well as on GC-2 cells in vitro, but does not have an effect on mRNA expression. All of these strongly indicate that ATF1 is a direct target of miR-34c.

In the present study, we have also developed an effective method to transfect the miR-34c inhibitor to spermatocytes in pubescent mouse testis. This is important for inspecting the specific function of miRNA *in vivo* by using transfection complexes containing liposomes and miRNA inhibitors injected into seminiferous tubules. In previous studies, many research groups applied seminiferous tubule injection plus electroporation methods to deliver plasmids to germ cells [36], [51], [52]. Here we use lipofection to deliver small molecules, such as the inhibitor of miR-34c. This method showed a relatively low toxicity and a high efficiency compared to electroporation within three days. Also, for short term research, this method has a lower cost and is more convenient and effective compared to knockout animals. The reason for the effectiveness of this method may be that primary spermatocytes assemble in the center of seminiferous tubules and near the lumen in pubescent mouse testis of 10 dpp∼18 dpp, and therefore, are relatively easily transfected. We choose mouse testis of 14 dpp when miR-34c's expression begin to rise as the injection time of transfection *in vivo*.

It is worth mentioning that transfecting double stranded small RNAs such as siRNA or miR-34c mimics, which exhibit a higher toxicity, lead to germ cell apoptosis *in vivo* 24 hours later than single-stranded small RNAs, such as the miR-34c inhibitors, when using the same amount of substance (50 pmol/µl). One explanation is that the toxicity of miRNA mimics or ATF1 siRNA-duplex is possibly associated with the off-target effects of double-stranded siRNAs, which may contribute to the final high toxicity of the cells [53]. Due to the relatively high toxicity of the short double-stranded siRNAs in the preliminary experiment *in vivo*, we did not analyze the ectopic expression of miR-34c and ATF1 siRNA in the testis model.

In conclusion, the present study suggests that miR-34c regulates germ cell viability in mouse testis through targeting ATF1. The miR-34c-ATF1 pathway is critical to the apoptosis of spermatocytes.

## Materials and Methods

### Animals and cell line

Adult male and female (6–8 month) Kunming white mice were purchased from the Animal Institute of the Chinese Medical Academy (Beijing, China) and were maintained under controlled light, temperature (22°c–24°c) and humidity (60–70%), with a 12-h light/dark cycle. One male and two female mice were put together to support breeding. The day the pups were born was marked as 1 dpp and the counting continued to the appropriate age for the experiments. All husbandry and experimental procedures were approved by the China Agricultural University Research Ethics Committee for Animal Sciences.

GC-2 germ cell line was kindly provided by Prof. Chunsheng Han of the Institute of Zoology, Chinese Academy of Sciences.

### MiR-34c detection by *in situ* hybridization(ISH)

MiR-34c ISH was carried out by using digoxigenin-labeled locked nucleic acid (LNA) probes. Hsa-miR-34c-5p miRCURY LNA microRNA detection probes and scrambled probes were purchased from Exiqon (Prod.No. 38542-00, Exiqon. Inc, MA, USA). We labeled the LNA probes with digoxingenin using a DIG oligonucleotide tailing kit (Roche) following the manufacturer's instructions, and the testis miR-34c ISH was performed as described by Song R. *et al* [54]. Briefly, mouse testes were collected at 13.5 dpc, 15.5 dpc, 17.5 dpc, 1 dpp, 7 dpp, 10 dpp, 12 dpp, 14 dpp, 16 dpp and at the adult stage. All of the pre pubertal testis samples were fixed in 4% PFA in DEPC PBS for 2 h. Adult testes were fixed overnight, and then placed in 30% sucrose overnight. The tissues were embedded in “Tissue Tek” O.C.T. compound (Takara) and 10 µm sections were cut using a cryostat. The slides were laid at room temperature (RT) for 30 min, fixed in 4% paraformaldehyde at RT for 10 min followed by two washes in PBS. The fixed sections were subjected to acetylation for 15 min and Protease K treatment for 5 min, which was followed by two PBS washes. The slides were then pre-hybridized for 8 h at RT and the hybridization was carried out at 50°c overnight. After stringency washing using 5×SSC for 10 min and 1×SSC for 1 hour at 60°c, the slides were incubated in a blocking solution for 1 h at RT, which was followed by incubation with AP-conjugated antibody to digoxingenin at 4°c overnight. After PBS and alkaline phosphates buffer washes, the slides were incubated in NBT and BCIP in the dark until the desired intensity of staining was reached.

### Preparation of cDNAs and analysis of real-time PCR

According to the protocols provided by the manufacturer, the total RNA of the testis was isolated using the Trizol Reagent (Vigorous Biotechnology Beijing Co., Ltd, China). MiR-34c expression assay were based on the method described by Chen C. *et al* [55]. U6 RNA was used for normalization of miRNA expression. Reverse transcriptase reactions contained the purified total RNA, 50 nM RT primer (the RT-34c stem-loop primer: CTCAACTGGTGTCGTGGAGTC GGCAATTCAGTTGA GGCAATCAG; or the U6 RT-primer: AACGCTTCACGAATTTGCGT). M-MLV Reverse Transcriptase (Promega, WI, USA) was used. The 15 µl reactions were incubated in a DNA Thermal Cycler 4800 for 30 min at 16°c, 30 min at 42°c and 5 min at 85°c and then stored at 4°c. All reverse transcriptase reactions included no-template controls and minus controls. Real-time PCR was performed using a standard Takara SYBR Premix Ex Taq protocol on an Applied Biosystems 7500 Real-time PCR System (Applied Biosystems). The real-time PCR primers used are as follows: miR-34c (Forward: GCTGCTGTAGGCAGTGTAGTTAG, Reverse: CTCAACTGGTGTCGTGGAGTC); U6 (Forward: CTCGCTTCGGCAGCACA, Reverse: AACGCTTCACGAATTTGCGT). The reactions were incubated in a 96-well plate at 95°c for 5 min, followed by 40 cycles at 95°c for 15 sec and 60°c for 1 min. All reactions were run in triplicate. The PCR product was confirmed to be miR-34c by sequencing. The melting curve in the PCR indicated a single product yield using this method. For real-time PCR analysis of ATF1 and apoptosis related genes, the following primers were used: ATF1 (Forward: CAGACGAGCAGCGGACAGT, Reverse: TGGCAGACGGCGTGGTA), Bcl-2 (Forward: GCTACCGTCGTGACTTCGC, Reverse: CTACCCAGCCTCCGTTATCC); Bcl-xL (Forward: ACCAGAC ACTGACCGTCCACT, Reverse: TGCTCCTGCCAGCCTCCAA); Bax (Forward: CCAGGATGCGTCCACCAAG, Reverse:GCAAAGTAGAAGAGGGCAACCAC); Bcl-w (Bcl2l2) (Forward: CTTTAGCAAACAGGAGCAGC, Reverse:AGGGCAGAACAGGTCACAA) Fas (Forward: AATCGCCTATGGTTGTTG, Reverse: TTCACGACTGGAGGTTCTA); GAPDH (Forward: GGTTGTCTCCTGCGACTTCA, Reverse: GGGTGGTCCAGGGTTTCTTA). The relative abundance of the genes was determined using the ABI PRISM 7500 equipped software (Applied Biosystems).

### MiR-34c inhibitor transfection *in vivo* via injection into seminiferous tubules

D14 male mice were anesthetized using Sodium Pentobarbital (8 µl of 0.5% solution/g bodyweight) injected intraperitoneally, and the testis was exposed under the dissecting stereomicroscope. 2′-Ome-miR-34c-5p inhibitor or control 2′-Ome-NC oligo (GenePharma Co.,Ltd, Shanghai, China) ranging from 1–2 µg and LipoFect2000 ranging from 0.5 µl–2 µl in 8 µl of HBS buffer (150 mM NaCl, 20 mM Hepes, pH 7.4), were used together with 0.04% Trypan Blue dye for monitoring the accuracy of the injection. The transfection mix, after filtrated through 0.22 µm Millipore filters, was injected into seminiferous tubules from the efferent duct using a glass injection pipette connected to an injector by a polyethylene tube. After the procedure, the skin was stitched and the mice were maintained for the following days.

### Cell culturing and transfection

GC-2 cells were cultured in Dulbecco's Modified Eagle Medium (DMEM) containing 10% fetal bovine serum (GIBICO) [56]. Transfections of ATF1 siRNA and miR-34c inhibitor or mimics (Shanghai GenePharma Co., Ltd, China) were performed using Lipofectamine 2000 (Invitrogen) according to the manufacturer's instructions. 24 hours after transfection, cells were harvested for real-time PCR, Western blot or cell apoptosis analysis. The following oligos were used to cell transfection: inhibitor NC (in-NC) (5′CAGUACUUUUGUGUAGUACAA3′); miR-34c inhibitor (5′GCAAUCAGCUAACUACACUGCCU3′); miR-34c mimics (Forward: 5′AGGCAGUGUAGUUAGCUGAUUGC3′ Reverse: 5′AAUCAGCUAACUACACUGCCUUU3′); stable negative control (s-NC) (Forward: 5′UUCUCCGAACGUGUCACGUTT3′ Reverse: 5′ACGUGACACGUUCGGAGAATT3′); ATF1 siRNA (Forward: 5′GACCUCUCUUCUGAAGAUAdTdT3′ Reverse: 5′UAUCUUCAGAAGAGAGGUCdTdT3′)

### Apototic Analysis

For TUNEL analysis, the testes were fixed in 10% formaldehyde and paraffin embedded. Paraffin sections were placed on positively-charged slides for *in situ* apoptosis detection using terminal deoxynucleotidyl transferase (TdT)-mediated deoxyribonucleotide triphosphate-digoxigenin nick-end labeling (TUNEL technique). Briefly, slides were incubated with 20 µg/ml Proteinase K for 15 min at room temperature and then washed in deionized water. Endogenous peroxidase was inactivated with 3% H_2_O_2_ in PBS for 5 min at room temperature. The subsequent staining was carried out according to the manufacturer's instructions (Promega, USA).

GC-2 cell apoptosis evaluation was performed by FITC-Annexin V/Propidium Iodide (PI) staining. Briefly, 1×10^5^ cells of each plate were collected and resuspended in 100 µl 1×Annexin V Binding Buffer. 2 µl FITC-Annexin V (BD Biosciences) were added and the cells were incubated at room temperature for 15 min in the dark. Then, we added PI to a final concentration of 5 µg/ml and incubated cells at room temperature for 5 min in the dark. Stained cells were analyzed as soon as possible (within 1 hr) via a FACSClibur flow cytometer and the CellQuest software (BD Biosciences, CA, USA). Annexin V+/PI− cells were considered to be early apoptotic cells and Annexin+/PI+ cells were determined as late apoptosis cells. GC-2 cell spontaneous apoptosis was performed 24 h after the siRNA or miRNA inhibitor transfection. For statistical analysis, more than 10000 total cells per sample were counted. The apoptotic index of GC-2 cells was expressed as the number of PI-positive cells per 100 cells.

### Western blot

To detect ATF1 protein levels in testis and GC-2 cells by Western blot analysis, the testes and harvested cells were lysed with cell lysis buffer (50 mM Tris-HCl, pH 7.4, 150 mM NaCl, 1% TritonX-100, 1% sodium deoxycholate, 0.1% SDS) containing 1 mM phenylmethanesulfonyl fluoride (PMSF). The protein concentration of each group was determined by using the BCA assay reagent (Vigorous Biotechnology, Beijing Co, Ltd.) according to the manufacturer's recommendations. Equal amount of proteins (50 µg) were electrophoresed on a 15% sodium dodecyl sulphate-polyacrylamide gel (SDS-PAGE) for ATF1 and GAPDH, and the bands were transferred to a polyvinylidene difluoride (PVDF) membrane (Bio-Rad Laboratories, Hercules, CA, USA). The membrane was blocked with 5% (w/v) non-fat dry milk in 0.05 M pH 7.4 Tris-buffered saline (TBS) for 3 h and incubated at 4°c overnight with a polyclonal mouse IgA anti-ATF1 (1∶500; Santa Cruz) or monoclonal mouse IgG anti-GAPDH (1∶10000; Ambion) in TBS. The PVDF membrane was then washed 3 times for 30 min in TBST (0.1% Tween-20 in TBS) and incubated for 2 h with a HRP-conjugated goat anti-mouse IgA (1∶1000) or goat anti-mouse IgG (1∶50000). After washing for 30 min with 3 changes of TBST, the membrane was treated with the SuperSignal West Pico kit (Thermo Scientific, USA) substrate at RT for 5 min. The relative intensity of each blot was assessed and analyzed. The intensity values pertaining to each group were normalized against the optical density of GAPDH corresponding to the same group within a single gel and expressed in terms of the means ± S.E.M of 3 independent experiments.

### Luciferase reporter assay

The dual-luciferase reporter genes were constructed using the psiCHECK™-2 vector (Promega, USA) and the 3′-UTR sequences of mouse ATF1. The ATF1 3′-UTR fragment cloning was done using over-lap PCR (Table 1). The sequences were introduced between the NotI and XhoI sites to Renilla luciferase 3′UTR. The Firefly luciferase vector was used for internal reference. Constructs with mutated 3′-UTR of ATF1 (Mut-ATF1) were used as negative controls. The 293T cells were cultured in DMEM supplemented with 10% fetal bovine serum. A total of 5×10^4^ cells per well were seeded in 24-well plates. After 24 h in culture, the cells were transfected using VigoFect (Vigorous Biotechnology Beijing Co., Ltd, China) with a mixture containing 200 ng/ml of the dual-luciferase reporter plasmid and 40 nM miR-34c mimics. Cells transfected with the Mut-ATF1 vector served as controls for normalization. Luciferase activity was measured by a Modulus™II microplate multimode reader (Promega) 24 h post-transfection using a Dual-Lucy Assay Kit (Vigorous Biotechnology Beijing Co., Ltd, China). All transfections were repeated independently at least three times.

### Statistical analysis

Results are presented as means ± S.E.M. Student's *t* test analysis was used for data analysis. A value of *p*<0.05 was considered to be statistically significant.
